# Preterm birth among the hmong, other Asian subgroups and non-hispanic whites in California

**DOI:** 10.1186/s12884-015-0622-0

**Published:** 2015-08-21

**Authors:** Zoua M. Vang, Irma T. Elo, Makoto Nagano

**Affiliations:** Sociology Department, McGill University, 855 Sherbrooke Street West, Montreal, Quebec H3A 2 T7 Canada; Sociology Department, University of Pennsylvania, 3718 Locust Walk, McNeil Building, Ste. 243, Philadelphia, PA 19104-6299 USA; Department of Obstetrics and Gynecology, McGill University, RI-MUHC, 1001 Decarie Blvd, Montreal, Quebec H4A 3 J1 Canada

## Abstract

**Background:**

We investigated very preterm (VPTB) and preterm birth (PTB) risk among Hmong women relative to non-Hispanic whites and other Asian subgroups. We also examined the maternal education health gradient across subgroups.

**Methods:**

California birth record data (2002–2004) were used to analyze 568,652 singleton births to white and Asian women. Pearson Chi-square and logistic regression were used to assess variation in maternal characteristics and VPTB/PTB risk by subgroup.

**Results:**

White, Chinese, Japanese, Korean, Asian Indian, and Vietnamese women had 36–59 % lower odds of VPTB and 30–56 % lower odds of PTB than Hmong women. Controls for covariates did not substantially diminish these disparities. Cambodian, Filipino and Lao/Thai women’s odds of VPTB were similar to that of Hmong women. But they had higher adjusted odds of PTB compared to the Hmong. There was heterogeneity in the educational gradient of PTB, with significant differences between the least and most educated women among whites, Chinese, Japanese, Asian Indians, Cambodians, and Laoians/Thais. Maternal education was not associated with PTB for Hmong, Vietnamese and Korean women, however.

**Conclusions:**

Studies of Hmong infant health from the 1980s, the decade immediately following the group’s mass migration to the US, found no significant differences in adverse birth outcomes between Hmong and white women. By the early 2000s, however, the disparities in VPTB and PTB between Hmong and white women, as well as between Hmong and other Asian women had become substantial. Moreover, despite gains in post-secondary education among childbearing-age Hmong women, the returns to education for the Hmong are negligible. Higher educational attainment does not confer the same health benefits for Hmong women as it does for whites and other Asian subgroups.

## Background

The Hmong population in the United States (US) numbered 205,355 in the year 2000 (authors’ calculations, 2000 Census, 5 % sample) [1].^1^ Reproductive age women (ages 15–44) made up a sizable segment of this population, representing 43 % of all women and 21 % of the total Hmong population. These women were mainly concentrated in three states: California (38 %; CA), Minnesota (23 %; MN) and Wisconsin (17 %; WI). Twenty-four percent of the women were born in the US while among the foreign-born, 94 % had immigrated to the US as children (under age 18). Thus at the dawn of the new millennium, most childbearing age Hmong women had spent a considerable amount of time in the US. Despite obstacles to education (notably high rates of poverty, early marriage and teen childbearing) [2, 3], Hmong women have made substantial educational gains [4]. Of Hmong women ages 18–44 in 2000, 24 % had earned a high school diploma, 19 % had some college education (including a 2-year Associates’ degree), and 6 % had obtained a bachelor’s degree or higher level of schooling (authors’ calculations, 2000 Census).

This profile of reproductive age Hmong women in the new millennium stands in stark contrast to the one in the 1980s, the decade immediately following the group’s mass migration to the US. In the 1980s, Hmong women were mainly refugees, had low educational attainment, high rates of poverty, high fertility, and births at both very young and older maternal ages [5–7]. These characteristics, combined with traditional cultural practices that purportedly discouraged use of Western health services (e.g., prenatal care) should have placed Hmong women at a high risk for poor birth outcomes and infant mortality [8, 9]. Yet, research from the 1980s showed that, contrary to expectations, birth outcomes of the Hmong were comparable to those of non-Hispanic whites (hereafter white). For example, in the 1980s the odds of having low birthweight and very low birthweight infants were similar among Hmong and white women [5] as were infant mortality rates [10].

Since the 1980s, a considerable amount of research has been conducted on infant health among Asian Americans and among different Asian subgroups in the US [11–16]. Most contemporary studies have omitted Hmong women from analysis altogether or aggregated them with Laotians and other Southeast Asians [17] with two exceptions. One recent study from CA documented that Hmong women, who gave birth between 2003–2005, had higher odds of low birthweight (OR 1.32, 95 % CI 1.18–1.49) and preterm birth (OR 1.49, 95 % CI 1.37–1.64) than white women, suggesting that birth outcomes among the Hmong have worsened relative to whites over time [18]. The second study using 2004–2008 birth record data from MN revealed a slightly higher, but not statistically significant, infant mortality rate among the Hmong than among other Asians (6.1 vs. 4.5 deaths per 1,000 births). Rates of low birthweight and preterm birth also did not significantly differ between Hmong women and the state average for Asians [19].

These recent studies beg additional questions about Hmong infant health. For one, we do not know how birth outcomes of contemporary Hmong women fare in comparison to other Asian subgroups since the reference groups in the two studies mentioned above are either whites or all Asians. Because there are substantial socioeconomic status (SES) differences among Asian subgroups in the US [20], it is important to contextualize Hmong birth outcomes not only vis-à-vis whites or all other non-Hmong Asians, but also in comparison to high SES (e.g., Chinese and Japanese) and low SES Asian ethnic groups (e.g., Cambodians). Second, maternal education is a strong predictor of birth outcomes, with higher education typically conferring a health advantage [21]. However, it is not known whether education is similarly protective against poor birth outcomes among Hmong women or in comparison to other Asian subgroups. Prior studies have shown that the relationship between maternal education and birth outcomes manifests differently for Hispanics [22] and black immigrants [23]; the same might be true for Asians as well. Furthermore, previous research has indicated that the relationship between maternal education and infant mortality also varies across Asian subgroups [14]; whether or not the association between maternal education and *birth outcomes* also differs by Asian ethnicity has not been examined.

In this paper, we investigate the risk of preterm birth among Hmong women relative to whites and other Asians in CA. We further assess whether the association between maternal education and preterm birth varies by maternal race/ethnicity. Preterm birth is a major public health concern, owing to the increased risk for infant mortality, implications for subsequent health, and high economic costs to society [24]. This study contributes to the growing literature on variation in birth outcomes among US racial and ethnic groups.

## Methods

We used 2002–2004 birth record data provided by the CA Department of Public Health, Center for Health Statistics. These records are based on the 1989 US Standard Certificate of Live Birth (CA did not implement the 2003 revised certificate until 2006). Information collected on the birth certificate includes maternal demographic characteristics, maternal medical conditions, and newborn characteristics [25, 26]. Birth certificate data has been shown to provide reliable information on maternal demographics, pregnancy history, prenatal care, delivery method, birth outcomes, and health insurance but low to moderate reliability for maternal behaviors during pregnancy (e.g., smoking, alcohol consumption), pregnancy/delivery complications, and medical conditions [26–28]. Ethics approval was provided by the Institutional Review Board of the University of Pennsylvania and the Committee for the Protection of Human Subjects (CPHS) at the CA Health and Human Services Agency. Informed consent from participants was waived by CPHS. CA has the largest concentration of Hmong Americans [29] and is one of the few states that collect detailed information on Asian ethnicity on the birth certificate, thereby enabling identification of births of Hmong women.

We identified 629,649 singleton live births to white and Asian women using information on the mother’s self-reported race/ethnicity. Asian women were categorized into nine subgroups: Hmong, Asian Indian, Chinese, Japanese, Korean, Vietnamese, Cambodian, Filipino, and Lao/Thai. We excluded 14,455 births (2 %) with implausible birthweight and gestational age combinations [30] and those with missing information on key explanatory variables (*n* = 46,542, 7 %). The analytical sample consisted of 568,652 births to white and Asian women.

We distinguished between very preterm (VPTB, <32 weeks) and preterm births (PTB, <37 weeks) based on last menstrual period (LMP) estimation of gestational age. Maternal characteristics included infant sex, nativity (US-born vs. foreign-born), age (<20, 20–29, 30–39, and 40+), education (< high school, high school diploma/GED certificate, some college, and college degree or higher), parity (0 previous live births, 1–2 previous live births, and 3+ previous live births), prenatal care (began in the 1st trimester, after 1st trimester, or no care/missing) and smoking during pregnancy (no vs. yes). We added two health conditions known to be prevalent among Hmong and other Asian women: preeclampsia (no vs. yes) and gestational diabetes (no vs. yes) [8, 16].

Rates of VPTB and PTB by race/ethnicity were calculated and odds ratios (OR) and 95 % confidence intervals (CI) were used to compare outcomes between Hmong and white and other Asian women. Logistic regression was used to compute adjusted odds ratios (aOR) to evaluate the association between race/ethnicity and VPTB/PTB, net of maternal characteristics. Hmong women served as the reference group for the descriptive and multivariate analyses because we are mainly interested in their risk of poor pregnancy outcomes vis-a-vis whites and other Asian subgroups.

We also examined whether the association between maternal education and PTB varied by mother’s race/ethnicity with the addition of interaction terms. All possible interactions between different race/ethnic categories and education levels were added simultaneously to the adjusted model. We assessed the statistical significance of the omnibus interaction effect using the likelihood ratio test [31]. Postestimation tests, with correction for multiple comparisons [32], were used to compare the least (<HS, HS) and most (College+) educated women within each race/ethnic group.^2^ Predicted probabilities were computed to facilitate interpretation of the interaction effects. The interaction analysis was restricted to PTB only because of empty cells for VPTB when race/ethnicity was interacted with education (e.g., there were no cases of Korean and Japanese mothers with less than high school education and VPTB). All models were estimated in STATA 13 [33].

## Results

Table 1 shows the distribution of maternal characteristics by race/ethnicity. Hmong women had a higher maternal risk profile than whites and other Asian subgroups. Nearly a quarter of Hmong births were to teen mothers. In contrast, only 5 % of white women and less than 5 % of women in other Asian subgroups (except Cambodian and Lao/Thai women) had children as teenagers. Low education was also prevalent among Hmong mothers: 30 % had less than a high school education whereas only 10 % had a college degree. Cambodian women were the only other group with similarly high rates of low education. Hmong women had the highest fertility, with 68 % of the women having had a previous live birth. In comparison, 45–58 % of white and other Asian women had one or more previous live births. Hmong women also had the lowest rate of early entry into prenatal care (61 %). Hmong women had lower rates of preeclampsia and diabetes compared to whites and other Asians, but this finding could be due to underreporting on the birth certificate given their later entry into prenatal care.

**Table 1:** Distribution of maternal characteristics by mother’s race/ethnicity, California, 2002–2004

	NH White	Hmong	Chinese	Japanese	Korean	Asian Indian	Vietnamese	Cambodian	Filipino	Lao/Thai	*p*-value
	(*N* = 415,800)	(*N* = 4,123)	(*N* = 33,982)	(*N* = 7,151)	(*N* = 13,190)	(*N* = 21,856)	(*N* = 24,396)	(*N* = 4,249)	(*N* = 39,497)	(*N* = 4,408)	
Nativity status, %											
Foreign-born (reference)	12.0	66.3	89.1	63.4	94.4	96.3	97.5	86.0	81.4	87.3	0.000
US-born	88.0	33.7	10.9	36.7	5.7	3.8	2.5	14.0	18.6	12.7	
Age, %											
<20	4.8	23.9	0.3	0.3	0.4	0.4	1.4	11.8	3.2	9.4	0.000
20–29 (reference)	41.8	56.7	23.2	19.8	29.0	53.8	36.0	51.1	39.5	52.6	
30–39	48.6	16.7	70.7	71.8	67.4	44.7	58.6	34.7	52.3	35.3	
40+	4.8	2.7	5.8	8.2	3.2	1.1	4.0	2.4	5.1	2.7	
Education, %											
Less than HS	6.4	30.0	6.3	0.9	0.7	3.4	13.9	28.2	3.8	16.4	0.000
HS diploma/GED	25.2	37.6	13.4	11.8	11.0	10.6	31.9	34.7	16.7	32.5	
Some college	24.9	22.8	14.8	25.0	17.9	18.8	20.1	22.9	34.2	23.9	
College degree or higher (reference)	43.5	9.6	65.4	62.4	70.5	67.2	34.1	14.3	45.3	27.2	
Parity, %											
0 (reference)	44.2	32.1	51.8	53.2	50.5	55.1	47.1	42.3	42.9	45.2	0.000
1–2	48.2	36.6	46.4	44.2	47.7	43.5	48.5	44.5	50.3	44.0	
3+	7.6	31.3	1.8	2.6	1.8	1.4	4.5	13.3	6.8	10.7	
Preeclampsia, %	1.5	0.2	0.4	0.7	0.5	0.6	0.7	0.5	1.5	0.7	0.000
Diabetes, %	1.9	0.9	3.6	2.5	1.9	4.2	4.0	1.6	4.4	2.7	0.000
Smoking during pregnancy, %	2.0	0.3	0.2	0.5	0.4	0.3	0.2	0.4	0.5	0.7	0.000
Prenatal care, %											
1st trimester (reference)	91.2	61.5	92.4	94.0	93.6	92.0	89.9	79.7	87.9	81.6	0.000
After 1st trimester	8.2	37.7	7.1	5.6	6.1	7.6	9.8	19.0	11.6	17.5	
No/missing	0.6	0.8	0.5	0.4	0.3	0.3	0.4	1.3	0.6	1.0	
Infant sex, %											
Male	51.3	51.6	52.0	51.6	51.7	51.8	51.0	51.3	51.8	51.4	0.126
Female (reference)	48.7	48.4	48.0	48.4	48.3	48.2	49.0	48.7	48.2	48.6	

*Note.* χ^2^ used to test differences in group proportions

Rates of VPTB and PTB by race/ethnicity are shown in Table 2. Unadjusted ORs and 95 % CIs comparing white/Asian subgroups to Hmong women are also shown in Table 2. The incidence of VPTB was low among all Asian subgroups; it was highest among Laotians/Thais (1.0 %) and lowest among Koreans (0.4 %). White, Chinese, Japanese, Korean, Asian Indian, and Vietnamese women had significantly lower odds of VPTB compared to Hmong women (ORs 0.41–0.64). Cambodian, Filipino, and Lao/Thai women had similar odds of VPTB as Hmong women.

**Table 2:** Rates (%) of VPTB and PTB by maternal race/ethnicity, and comparison of VPTB and PTB with Hmong women, California, 2002–2004

	Incidence (%) of		HMONG VS. WHITES/ASIAN SUBGROUPS			
	VPTB	PTB	VPTB		PTB	
	(<32 weeks)	(<37 weeks)	OR	95 % CI	OR	95 % CI
White	0.60	5.43	**0.61**	**(0.45, 0.84)**	**0.64**	**(0.57, 0.72)**
Hmong	0.97	8.22	(reference)	---	(reference)	---
Chinese	0.49	4.69	**0.50**	**(0.36, 0.71)**	**0.55**	**(0.49, 0.62)**
Japanese	0.56	5.10	**0.57**	**(0.37, 0.89)**	**0.60**	**(0.52, 0.70)**
Korean	0.40	3.79	**0.41**	**(0.27, 0.62)**	**0.44**	**(0.38, 0.51)**
Asian Indian	0.63	5.53	**0.64**	**(0.45, 0.92)**	**0.65**	**(0.58, 0.74)**
Vietnamese	0.51	5.86	**0.52**	**(0.36, 0.75)**	**0.70**	**(0.61, 0.79)**
Cambodian	0.92	9.91	0.95	(0.61, 1.47)	**1.23**	**(1.06, 1.43)**
Filipino	1.00	8.14	1.03	(0.74, 1.42)	0.99	(0.88, 1.11)
Lao/Thai	1.02	9.07	1.05	(0.69, 1.62)	1.11	(0.96, 1.30)

*Note*. Bold figures indicates statistically significant differences at *p* < 0.05 level

PTB rates were highest among Cambodians (9.9 %), but lowest among Koreans (3.8 %). PTB was also significantly lower among white, Chinese, Japanese, Korean, Asian Indian, and Vietnamese women than Hmong women (ORs 0.44–0.70). Differences in PTB between Hmong, Filipino and Lao/Thai women were trivial, whereas the odds of PTB for Cambodian women was 1.23 times higher than for Hmong women.

Adjusted ORs from logistic regressions predicting VPTB and PTB are shown in Table 3. The addition of maternal characteristics did not attenuate the gap in VPTB risk for Hmong women relative to white, Chinese, Japanese, Korean, and Vietnamese women. In contrast, Hmong and Asian Indian women had similar odds of VPTB after controlling for maternal characteristics.

**Table 3:** Comparison of Hmong women to whites and other Asian subgroups with multivariate logistic regression (*N* = 568,652): CA, 2002–2004

	VPTB	PTB
	aOR (95 % CI)	aOR (95 % CI)
Mother’s race/ethnicity:		
Hmong	1.00	1.00
White	**0.59 (0.42, 0.81)**	**0.70 (0.62, 0.78)**
Chinese	**0.56 (0.39, 0.79)**	**0.68 (0.60, 0.77)**
Japanese	**0.59 (0.38, 0.93)**	**0.72 (0.62, 0.84)**
Korean	**0.49 (0.32, 0.75)**	**0.58 (0.50, 0.67)**
Asian Indian	0.81 (0.56, 1.16)	**0.87 (0.76, 0.99)**
Vietnamese	**0.54 (0.37, 0.77)**	**0.80 (0.70, 0.91)**
Cambodian	0.91 (0.59, 1.43)	**1.30 (1.11, 1.51)**
Filipino	1.10 (0.79, 1.53)	**1.17 (1.04, 1.32)**
Lao/Thai	1.08 (0.70, 1.67)	**1.25 (1.07, 1.45)**

*Notes*. Bold figures indicates statistically significant differences at *p* < 0.05 level. Models adjusted for infant sex, maternal age, education, nativity status, parity, preeclampsia, diabetes, smoking during pregnancy, and prenatal care

Adjustment for covariates explained some of the difference in PTB risk between Hmong women and white, Chinese, Japanese, Korean, Asian Indian, and Vietnamese women (aORs 0.58–0.70). For example, the unadjusted odds of PTB was 56 % lower for Korean than Hmong women (OR 0.44, CI 0.38–0.51; Table 2), but this gap was reduced to 42 % (aOR 0.58, CI 0.50–0.67; Table 3) in the adjusted model. Despite the attenuated gap in PTB, Hmong women remained significantly more likely to deliver a premature infant than women from these other Asian subgroups. In contrast, Filipino and Lao/Thai women’s odds of PTB became significantly higher than that of Hmong women in the adjusted model (aOR1.17, CI 1.04–1.32 and aOR 1.25, CI1.07–1.45, respectively), and Cambodian women continued to have significantly higher odds of PTB than Hmong women (aOR 1.30, CI 1.11–1.51).

Finally, we assessed whether the association between maternal education and PTB varied between the Hmong and whites and the other Asian subgroups with the addition of interaction terms. The likelihood ratio test showed a statistically significant omnibus interaction effect (LR ^2^(27) = 42.60, *p*-value = 0.029). Postestimation tests comparing the least (<HS, HS) and most (College+) educated women by maternal racial/ethnicity revealed statistically significant educational gradients for whites, Chinese (HS vs. College + only), Japanese (HS vs. College + only), Asian Indians (HS vs. College + only), Cambodians, Filipinos, and Laotians/Thais (Table 4). The most educated women from these subgroups had the lowest odds of PTB whereas their least educated counterparts had the highest. In contrast, Hmong, Vietnamese and Korean women had relatively flat gradients. The odds of PTB were just as high for the most educated Hmong, Vietnamese and Korean women as it was for their least educated counterparts.

**Table 4:** Postestimation tests for contrasts between the least (<HS, HS) and most (College+) educated women by race/ethnicity: CA, 2002–2004

	COMPARISON			
	<HS vs. College+		HS vs. College+	
	OR (Bonferroni 95 % CI)	Bonferroni *p*-value	OR Bonferroni 95 % CI)	Bonferroni *p*-value
Mother’s race/ethnicity				
Hmong	1.19 (0.60, 2.35)	1.000	1.18 (0.60, 2.31)	1.000
White	1.70 (1.56, 1.85)	0.000	1.32 (1.25, 1.40)	0.000
Chinese	1.33 (0.99, 1.80)	0.078	1.36 (1.09, 1.70)	0.000
Japanese	1.09 (0.18, 6.63)	1.000	1.60 (1.01, 2.54)	0.041
Korean	1.55 (0.38, 6.32)	1.000	1.44 (0.96, 2.17)	0.140
Asian Indian	1.48 (0.95, 2.29)	0.142	1.37 (1.04, 1.81)	0.011
Vietnamese	1.24 (0.96, 1.60)	0.205	1.11 (0.90, 1.36)	1.000
Cambodian	1.95 (1.07, 3.54)	0.014	1.96 (1.09, 3.52)	0.010
Filipino	1.47 (1.12, 1.92)	0.000	1.22 (1.04, 1.43)	0.003
Lao/Thai	1.74 (1.05, 2.90)	0.019	1.78 (1.14, 2.78)	0.002

*Notes*. Postestimation tests were computed for a total of 20 contrasts between the two least educated groups (<HS, HS) and the most educated group (College+). Contrasts of marginal linear predictions were computed in Stata using the *contrast* command. *P*-values and 95 % confidence intervals were adjusted for multiple comparisons with the Bonferroni method using the *mcompare* option

*Notes*. Statistically significant educational gradients of PTB were observed for white, Chinese (HS vs. College + only), Japanese (HS vs. College + only), Asian Indian (HS vs. College + only), Cambodian, Filipino, and Lao/Thai women. Predicted probabilities estimated from fully adjusted model, including interaction terms between maternal education and race/ethnicity

**Fig. 1:**
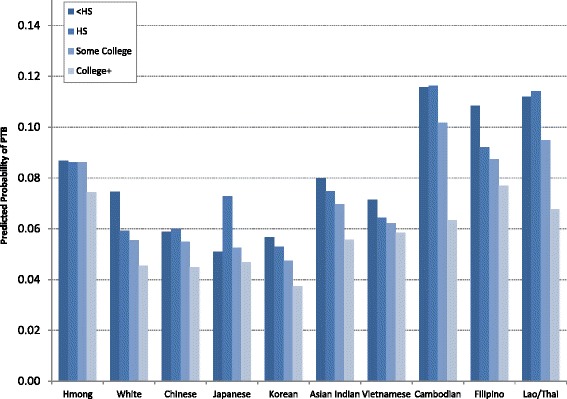
Predicted probability of PTB by mother’s education and race/ethnicity (*N* = 568,652): CA, 2002–2004

Predicted probabilities of PTB by mother’s education and race/ethnicity are shown in Fig. 1. Noteworthy among the distributions is the steeper education gradients for Cambodian and Lao/Thai women. The difference in PTB between the least and most educated women in these two subgroups was quite large. In comparison, gradients for white, Chinese, Asian Indian, and Filipino women were also statistically significant but relatively flatter. Hmong women’s flat education gradient appears to be the result of the better performance of the poorly educated. The probabilities of PTB for poorly educated (<HS, HS) Hmong women were much lower than for poorly educated Cambodian, Filipino, and Lao/Thai women. At the same time, the attainment of a four-year college degree does not appear to confer the same health benefits to Hmong women as it does for whites and (most) East Asians. The probability of PTB for highly educated Hmong women was nearly double that of white, Chinese, Japanese, and Korean women.

## Discussion

Studies dating back to the 1980s found no differences in adverse birth outcomes between Hmong and white women [5, 10]. By the early 2000s, however, the disparities in VPTB and PTB between Hmong and white women, as well as between Hmong and other Asian women had become substantial. White, Chinese, Japanese, Korean, Asian Indian and Vietnamese women had between 36–59 % lower odds of VPTB and between 30–56 % lower odds of PTB than Hmong women. Controls for maternal characteristics did not substantially diminish these disparities, with the exception of VPTB between the Hmong and Asian Indians. The odds of VPTB among Cambodian, Filipino and Lao/Thai women were similar to that of Hmong women, but these three subgroups had higher adjusted odds of PTB.

Previous research found better birth outcomes for East Asian-origin (e.g., Chinese, Japanese, and Korean) and Vietnamese women compared to white women [12] but worse outcomes among Cambodian, Laotian and Filipino women relative to white [12], Japanese [14], or Chinese [16] women. Our results for the Hmong corroborate the higher risk of adverse birth outcomes among Southeast Asians relative to whites and East Asians. Our analysis further reveals that within the higher risk Southeast Asian population, the Hmong are not the worst off subgroup. Indeed Hmong women appear to be less susceptible to PTB than Cambodian, Lao/Thai or Filipino women once group differences in maternal characteristics are taken into account.

Adjustment for known maternal risk factors available on the birth certificate (e.g., age, education, prenatal care, preeclampsia and diabetes) were insufficient to explain the higher risk of VPTB and PTB between the Hmong and whites, Chinese, Japanese, Koreans, Asian Indians (PTB only) and Vietnamese. The Hmong’s lower PTB risk relative to Cambodians, Laotians/Thais and Filipinos also remained unexplained. Ethnic variations in birth outcomes among Asian women have been attributed to differences in the socioeconomic environments of source countries, given that most Asian mothers are foreign-born [14]. In our sample more than 80 % of Chinese, Korean, Asian Indian, Vietnamese, Cambodian, Filipino, and Lao/Thai women were foreign-born. The proportions of foreign-born among Hmong and Japanese women were much lower (66 % and 63 %, respectively). Thus, while source country environmental differences may be relevant for some Asian subgroups in our study, pre-migration factors are less applicable to the Hmong and Japanese.

Other researchers have pointed out the need for “new risk models” in assessing Asian birth outcomes that extend beyond traditional obstetric risk factors such as maternal age, parity, and prenatal care use [12]. We similarly agree that maternal sociodemographic characteristics and prenatal care alone do not adequately capture the complexity of risk environments that Hmong and other Asian women are exposed to in the US. Research needs to incorporate post-migration factors such acculturation, social support, and structural inequality (e.g., discrimination) in analyses of Asian birth outcomes. For example, the weathering hypothesis suggests that cumulative effects of stressors associated with long-term socioeconomic disadvantage and discrimination can negatively impact pregnancy outcomes through behavioral and physiological pathways [34]. Weathering may be particularly relevant for Hmong women given that they have the highest poverty rate among Asians in the US [20] and Hmong people encounter both interpersonal discrimination in their interactions with out-group members [35] as well as institutional discrimination in the healthcare system [36].

We also found a flat education gradient for Hmong, Vietnamese and Korean women. This finding is inconsistent with research that has typically documented a strong association between maternal education and birth outcomes, including PTB [37–39]. The finding is, nevertheless, consistent with studies that have documented weaker education gradients for other minority groups, e.g., Hispanics [22] and black immigrants [23]. Different socioeconomic gradients in the origin countries, health selection, and sociocultural factors have been suggested as possible explanations for the attenuated SES health gradient among Hispanic subgroups [22]. Considering that Hmong, Vietnamese and Koreans have very different migration and settlement histories, cultures, and occupy different socioeconomic positions in US society, health selection and origin country conditions may be insufficient to explain why such dissimilar groups would display a similar pattern of attenuated educational effects on PTB. Possible answers may lie in sociocultural factors.

For example, the flat education gradient for Hmong women may be related to the health behaviors of less educated mothers. Research indicates that the odds of delayed prenatal care is significantly higher among less educated (high school diploma or less) Hmong women than those with at least some college education [40]. It is possible that educational differences in access to and utilization of prenatal care may not translate into differential PTB risk because of a culturally-linked preference for self-care during pregnancy among less educated Hmong women [9]. Thus, despite the underutilization of structured prenatal care [40, 41] less educated Hmong women may nonetheless receive health benefits from their traditional self-care practices, thereby diminishing the benefits typically associated with higher educational attainment.

Our study is not without limitations. There are a couple of issues related to the generalizability of our results. First, our findings may not be generalizeable to Hmong, Asian and white women living elsewhere in the US. Future research should examine Hmong women living in other states with large Hmong populations (e.g., MN and WI) in order to provide a national profile of birth outcomes among the Hmong. Second, the generalizability of our findings to *contemporary* US Hmong women could be questioned, especially if the profile of reproductive age Hmong women has changed substantially since 2000. In additional analysis not shown, we generated a comparable profile of reproductive age Hmong women for 2012 (authors’ calculations, 2012 American Community Survey 3-year sample—the most recent, reliable data available) [1] and found differences in two characteristics: the proportion of native-born women and the proportion of women with higher education. The proportion of native-born women increased by 30 % between 2000 and 2012. Given that foreign-born women tend to have better birth outcomes than their native-born counterparts [42], the greater share of native-born Hmong women today might increase the Hmong’s PTB risk relative to whites and other Asian subgroups. Since nativity status is not associated with PTB for Hmong women in our sample, however, the increase in this parameter is unlikely to affect our findings. The proportions of women with some college and a college degree or higher also increased from 20–42 % and from 6–14 %, respectively, between 2000 and 2012. In this regard, we note that Vietnamese women —a group with a large proportion of college-educated women (34 %)—exhibited a flat education gradient. Considering that only 14 % of reproductive age Hmong women in 2012 have a college degree, the growth in the proportion of educated Hmong women may not be large enough to affect our current findings. Further research with more recent data should clarify this issue.

There are several issues related to data quality, which might affect our results. First, there may have been some misclassification of Hmong births as Laotian because of underreporting of Hmong ethnicity on the birth certificate. Research based on the 2010 Census indicates that some Hmong respondents reported a Laotian as opposed to a Hmong ethnic identity [29]. Misclassification of Hmong births would result in underestimated VPTB/PTB rates for Hmong women in our study and potentially overestimate the risk of adverse outcomes among Laotian women. Second, variation in data collection across different hospitals [26],^3^ reliance on maternal recall [43] and differential reporting by race/ethnicity or English language proficiency may compromise the reliability of birth certificate data [27]. We know of no studies which indicate that Asians and whites—and different Asian subgroups—systematically differ from one another in the reporting of birth record information. But given the high proportions of immigrants among some of our Asian subgroups, language-related underreporting for variables known to have low to moderate reliability (e.g., pregnancy-related medical conditions) may be problematic [28]. For example, measurement error in gestational diabetes and preeclampsia could result in biased estimates of their effects on PTB. However, overall findings related to race/ethnic disparities in PTB may be unaffected since the reliability of birth outcome variables is high [26, 27]. Lastly, estimates of VPTB/PTB could be biased because LMP-estimated gestational age is prone to measurement error, particularly for preterm births [44, 45]. We corrected for possible measurement errors in our dependent variable by omitting births with implausible birth weight-gestational age combinations, a strategy also used in prior studies [46]. Birth record data is widely used in the US to study race/ethnic variation in birth outcomes and are the only data available to study PTB risk among small race/ethnic subgroups.

Additional limitations include limited information available on the birth certificate which precludes a thorough examination of factors that may contribute to the observed race/ethnic differences in VPTB/PTB. We were also unable to account for the clustering of multiple births to the same woman, which would result in underestimated standard errors. This issue may be especially relevant for Hmong women given their high fertility. Despite these limitations our study makes a valuable contribution to the small but important body of research on infant health among Hmong women in the US.

## Conclusions

In conclusion, we found that Hmong women had higher risks of VPTB and PTB relative to white, East Asian, Asian Indian (PTB only), and some Southeast Asian (Vietnamese only) women. Birth outcomes among Hmong women are an important public health issue that has not received attention in recent studies of infant health outcomes among Asians in the US. Given that early-in-life health conditions can have enduring effects across the life course the ethnic variations in birth outcomes we observed may have long-term consequences for racial/ethnic health disparities and public health.

## Endnotes

^1^Hmong individuals were identified in the 2000 Census using a combination of (detailed) race, (first and second) ancestry, and language. This broad definition allowed for the inclusion of Hmong persons of mixed race/ethnic background (e.g., Mexican-Hmong Americans).

^2^Once we determined that the omnibus interaction effect was statistically significant with the likelihood ratio test, postestimation tests contrasting the least (<HS, HS) and most (College+) educated women within each race/ethnic group were computed. These educational categories were chosen for the comparison because race/ethnic variation in infant health between the poorest and best educated women is indicative of differential returns to education [23]. Moreover, preliminary analysis showed no differences between the least (<HS, HS) and second most educated (some college) subgroups for all women in our data, except whites. The *contrast* command in Stata was used to compute the education contrasts within each race/ethnic group. To reduce the chances of false positives (type I error) stemming from multiple comparisons, we used the *mcompare* option in Stata to produce Bronferroni-adjusted 95 % confidence intervals and *p*-values.

^3^Physicians and other hospital staff are responsible for completing or verifying information on the birth certificate [26]. Staff also relies on maternal recall for some types of information (e.g., pregnancy history) [43]. Data are usually gathered within the first few days of delivery and then transmitted electronically to the state’s bureau of vital statistics for processing [26].
